# Supplementary material for: Turtles in Malaysia: A Review of Conservation Status and a Call for Research
Source: Animals (Basel). 2022 Aug 25;12(17):2184. doi: 10.3390/ani12172184 (PMC9454601; doi:10.3390/ani12172184)
Supplement: Supplementary file 1 [file animals-12-02184-s001.zip › animals-1827249-supplementary.pdf]

Supplementary Table

Supplement Table S1. The list of turtle types in Malaysia.

New World Turtle	Land Tortoise	Sea Turtle	Soft Shell Turtle	Hard Shell Turtle
Trachemys scripta	*Manouria emys*	*Chelonia mydas*	*Amyda cartilaginea*	*Batagur affinis*
	Manouria impressa	*Dermochelys coriacea*	*Chitra chitra*	*Batagur borneoensis*
		Eretmochelys imbricata	*Chitra indica*	*Cuora amboinensis*
		Lepidochelys olivacea	*Dogania subplana*	*Cyclemys dentata*
			Pelochelys cantorii	*Heosemys annandalii*
			Pelodiscus sinensis	*Heosemys spinosa*
				Indotestudo elongata
				Malayemys macrocephala
				Notochelys platynota
				Orlitia borneensis
				Siebenrockiella crassicollis
